# Development and Preclinical Investigation of Physically Cross-Linked and pH-Sensitive Polymeric Gels as Potential Vaginal Contraceptives

**DOI:** 10.3390/polym14091728

**Published:** 2022-04-23

**Authors:** Ankit Rochani, Vivek Agrahari, Neelima Chandra, Onkar N. Singh, Timothy J. McCormick, Gustavo F. Doncel, Meredith R. Clark, Gagan Kaushal

**Affiliations:** 1 Department of Pharmaceutical Science, Thomas Jefferson University, Philadelphia, PA 19107, USA; ankit.rochani@jefferson.edu; 2 CONRAD, Eastern Virginia Medical School, Norfolk, VA 23507, USA; vagrahari@conrad.org (V.A.); chandrn@evms.edu (N.C.); onsingh2@yahoo.com (O.N.S.); tmccormick@conrad.org (T.J.M.); doncelgf@evms.edu (G.F.D.); mclark@conrad.org (M.R.C.)

**Keywords:** contraceptive formulation, cross-linked gel, rheology, physical barrier, vaginal sperm transport

## Abstract

This study explored the development of cross-linked gels to potentially provide a physical barrier to vaginal sperm transport for contraception. Two types of gels were formulated, a physically cross-linked iota-carrageenan (C_i_) phenylboronic acid functionalized hydroxylpropylmethyacrylate copolymer (PBA)-based (C_i_-PBA) gel, designed to block vaginal sperm transport. The second gel was pH-shifting cross-linked C_i_-polyvinyl alcohol-boric acid (C_i_-PVA-BA) gel, designed to modulate its properties in forming a viscoelastic, weakly cross-linked transient network (due to C_i_ gelling properties) on vaginal application (at acidic pH of ~3.5–4.5) to a more elastic, densely cross-linked (due to borate-diol cross-linking) gel network at basic pH of 7–8 of seminal fluid, thereby acting as a physical barrier to motile sperm. The gels were characterized for dynamic rheology, physicochemical properties, and impact on sperm functionality (motility, viability, penetration). The rheology data confirmed that the C_i_-PBA gel was formed by ionic interactions whereas C_i_-PVA-BA gel was chemically cross-linked and became more elastic at basic pH. Based on the screening data, lead gels were selected for in vitro sperm functionality testing. The in vitro results confirmed that the C_i_-PBA and C_i_-PVA-BA gels created a barrier at the sperm-gel interface, providing sperm blocking properties. For preclinical proof-of-concept, the C_i_-PBA gels were applied vaginally and tested for contraceptive efficacy in rabbits, demonstrating only partial efficacy (40–60%). Overall, the in vitro and in vivo results support the development and further optimization of cross-linked gels using commercially available materials as vaginal contraceptives.

## 1. Introduction

The unmet need for contraception remains a global health priority. The World Health Organization (WHO) estimates that 214 million women of reproductive age in developing countries who want to avoid pregnancy are not using contraceptive methods [1]. The major obstacles include lack of information, access, safety concerns, and limited choice of contraceptive methods differing in site of administration, effectiveness, and duration of use [2]. These methods include hormones (e.g., oral pills, syringeable, implants), intrauterine devices (IUDs), sterilization (e.g., tubal ligation), chemical barriers (e.g., spermicides), and physical barriers (e.g., male and female condoms, cervical caps, diaphragms) [3]. Barrier contraceptives are intended to prevent fertilization by physically or chemically blocking the sperm and egg interaction. Of these, chemical barriers represent a viable method; however, only nonoxynol-9 (N-9) is currently on the market, but it causes side effects with prolonged use [4]. The physical barrier-based female contraceptives are non-hormonal, safe, can be used without seeking a health care provider, and effective if used correctly. However, the disadvantages include the requirement of a high degree of consistent and correct use before each act of sexual intercourse, incidence of allergic reactions and urinary tract infections, and chances of slippage and breakage during coitus, which necessitate a back-up use of emergency contraception. These limitations require the development of alternative means of physical barrier contraceptives that provide ease of application, retention during coitus, discreet, and on-demand use. Thus, the choice of delivery system and biomaterials, which can provide flexible properties for ease of application, safety, and durability to withstand the shear forces of coitus, is important.

Vaginal delivery systems for contraception include a variety of dosage forms [5] and one of the most widely used are gels, owing to their capability of adhesion to surfaces for a reasonable period and the feasibility of low-cost manufacturing. The vaginal contraceptive gels are specifically developed as spermicides, but to date, not much work has been undertaken to develop gel products that can act as an on-demand stimuli-responsive physical barrier to sperm transport to the uterus. The synthesis and characterization of a pH-responsive synthetic mucin-like polymer (SMP) gel has been proven to inhibit viral transport in vitro, at both acidic and neutral pH [6,7]. The SMP gel is formulated using two hydroxypropyl methacrylamide (HPMA)-based copolymers, each functionalized with phenylboronate (PBA) or salicylhydroxamate (SHA) vinyl moieties [8]. Because of the pH-sensitivity of PBA-SHA copolymer’s reversible covalent cross-linking chemistry [8,9], the polymer network transitioned from a more fluid, lubricating, gel-like consistency at acidic vaginal pH to that of a densely cross-linked physical barrier to the virus when exposed to basic pH of semen fluid. The PBA-SHA cross-linking works by boronic acids or the boronate species undergoing a reversible condensation reaction with cis-diols to form cyclic boronate esters. The complex formed between PBA and SHA exists in the tetrahedral boronate conformation and yields a viscoelastic, cross-linked network at acidic pH. The proof-of-concept (POC) that SMP gel can prevent sperm transport has been reported in animal models [10]. Though innovative, a high concentration of each of the polymers (~200 mg/mL) was used in formulating the SMP gel to provide 100% contraceptive efficacy, which adds to the cost and manufacturing complexity in requiring large quantities of the custom polymers for bulk production of the gel. All these challenges support exploration of other commercially available polymers in generating the vaginal gels as a physical contraceptive barrier to sperm.

Previous studies showed that biomaterials such as polyvinyl alcohol (PVA) and polysaccharides could chemically cross-link with boric acid (BA) or BA-derivatives in developing soft biodegradable hydrogels [11,12,13]. PVA is a biodegradable polymer that has been extensively explored in various forms of delivery systems, including gels [14,15,16]. The anionic polysaccharides (e.g., carrageenan and hyaluronic acid) have also been explored for hydrogel preparations due to their characteristic ionic interactions [17]. Studies have also reported the use of carrageenan-iota (C_i_) vaginal hydrogel formulations for contraceptive applications [18,19,20]. C_i_ also has a natural gelling behavior in the presence of monovalent cations and forms soft, syringeable hydrogels [21]. Thus, carrageenan is not only safe but can also serve as an ionic gelling agent to BA or BA-derivatives [22]. Hence, the composite materials of natural polysaccharides, polymers, and BA derivatives could provide potential alternatives in developing vaginal hydrogel formulations.

The purpose of this work is to show POC development of vaginal contraceptive formulations using primarily commercially available pharmaceutical polymers such as carrageenan and PVA, cross-linkers, and excipients to provide a physical barrier to sperm transport. This includes designing formulations that can modulate its properties in forming a viscoelastic, weakly cross-linked, transient network on vaginal application, transitioning to a more elastic and densely cross-linked network in the presence of semen. SMP gel was developed as a control barrier gel and its physicochemical and rheological properties were considered as baseline parameters for developing the gel formulations as potential physical contraceptive barriers in this study. For POC evaluation, two formulations were designed: (a) a physically cross-linked polymeric gel targeted to remain in the vagina after the application and to block sperm transport in the presence of seminal fluid, and (b) a biologically inspired pH-sensitive chemically cross-linked gel that because of the cross-linking chemistry of the polymer network, can transition from a more fluidic, lubricating, gel-like consistency at acidic vaginal pH to a densely cross-linked physical barrier to sperm when exposed to a basic pH of seminal fluid. Both of these gels were characterized in vitro (dynamic rheology, physicochemical properties, and sperm functionality) and the selected lead gels were tested in vivo for contraceptive efficacy in rabbits.

## 2. Materials and Methods 

### 2.1. Materials

Carrageenan (iota type: C_i_) polymer was provided by AEP Colloids, Division of Sarcom, Inc. (Hadley, NY, USA). Polysciences, Inc. (Warrington, PA, USA) synthesized and provided the PBA and SHA polymers copolymerized with a 2-hydroxypropyl methacrylamide (pHPMA) polymer backbone with ~9% and 5% PBA and SHA functionalities, respectively. Sodium acetate, PVA, and BA were obtained from Sigma-Aldrich (St. Louis, MO, USA). Sodium hydroxide (NaOH), hydrochloric acid (HCl), parabens (methyl and propyl), and glycerol were obtained from Fisher scientific (Waltham, MA, USA). 

### 2.2. Methods

#### 2.2.1. Formulation of Physically Cross-Linked C_i_-PBA Gels 

The physically cross-linked gel was prepared using PBA-HPMA co-polymer and commercially available C_i_ polymer at different concentrations to understand their cross-linking behavior. Specific quantities of PBA-HPMA and C_i_ polymers were separately dissolved in 25 mM sodium acetate buffer (pH~4.2). The PBA-HPMA solution was kept overnight under slow stirring for the complete solubilization of the polymer and once a clear solution was obtained, it was titrated with 1N NaOH to pH~4.2. The C_i_ polymer at different concentrations was dissolved with heating at 80–90 °C under constant stirring for ~30 min to generate homogenous and clear solutions. The pH of these solutions was adjusted to ~4.2 using 1 N HCl. The volume ratio of mixing the PBA-HPMA and C_i_ solutions was 1:1 v/v with adding additional 1 mL of acetate buffer in the mixed sample. This solution was kept overnight at room temperature to allow complete polymerization in order to enhance the flowability and homogeneity of the C_i_-PBA gels. The gel samples were adjusted to acidic (pH 3.5–4.5) or basic (pH 7–8) pH using HCl or NaOH solutions, respectively. As a control, the PBA-HPMA and SHA-HPMA polymers-based SMP gel at final concentration of 100 mg/mL for each polymer and pH 3.8 was formulated using a previously published method [9]. Paraben-based preservatives (methyl and propyl parabens at a combined concentration of 0.27% w/w) in glycerol (2.5% w/w) were added as excipients to make the final gel product. The prototype formulations were then evaluated for physicochemical properties, dynamic rheology (elastic (G′), and viscous (G″) moduli as a function of angular frequency (ω)), osmolality, homogeneity, syringeability, self-healing/spinnbarkeit behavior, and gelling capability at acidic pH (~4–4.5) and neutral to basic pH of ~7–8. The rationale for selecting these pH ranges was based on the fact that the normal cervicovaginal fluid is acidic (pH~3.5–4.5) and becomes neutral to basic (pH~7–8) in the presence of seminal fluid during sexual intercourse [23,24].

#### 2.2.2. Formulation of Chemically Cross-Linked C_i_-PVA-BA Gels

The chemically cross-linked, pH-sensitive gels were formulated using PVA and C_i_ polymers in combination with BA as a cross-linker. Separate solutions of PVA and BA were prepared in DI water and heating to 80–90 °C with constant stirring for ~30 min. Both solutions were allowed to cool down, mixed and gently vortexed, and kept at room temperature for complete solubilization for another 2 h. Once the PVA-BA solution became homogeneous and clear, specific amounts of C_i_ (prepared per the method in Section 2.2.1) were added. The solutions were gently mixed and kept in a water bath at 60 °C for another 10–15 min to make them homogenous and clear and then left overnight at room temperature for complete polymerization and generation of chemically cross-linked C_i_-PVA-BA gels. 

### 2.3. Rheological Measurements

Rheological measurements of the gels were made using an AR-G2 rheometer (TA Instruments, Wilmington, DE, USA). A frequency sweep experiment was performed to examine the nature and viscoelastic behavior of the synthesized gels at two temperatures (25 °C and 37 °C) and pH conditions (~4–4.5 and 7–8). The measurements were taken using plate SST ST 20 mm (zero degree) Smart Sweep geometry (Part No. 511200.905, Serial No. 108064). The rheometer was calibrated as per the standards provided by TA Instruments. For the measurements, ~750 µL of each gel was placed on the sample stage of the instrument and the frequency sweep range of 0–100 rad/s and 5% strain were applied.

#### 2.3.1. Storage (G′) and Loss (G″) Modulus and Tan Δ Measurements

The G′ and G″ values obtained from the frequency sweep run were plotted to understand the effects of concentration, temperature (25 °C and 37 °C), and pH (acidic and basic) on the structural properties of gels. The data were presented as overlay plots for the gels. The frequency sweep data were also used to evaluate the tan delta (tan Δ) values, defined as the ratio of G′ and G″. In general, tan Δ values > 1 show liquid-like material, and values of <1 or close to zero confirm the viscoelastic nature of the gels [25,26].

#### 2.3.2. Complex Viscosity

In addition to G′ and G″ modulus, complex viscosity was also recorded for the gels on rad/s scale. The overall resistance of the material to deformation regardless of the storage or deformation loss was recorded as complex modulus, whereas the ratio of complex modulus to angular frequency (ω) was recorded as complex viscosity [27]. Complex viscosity helps in understanding the viscosity of materials under oscillatory stress. The data curves were fitted into the standard models provided in the Trios software (TA Instruments) to calculate the zero-shear-rate viscosity.

### 2.4. Creep Recovery Analysis 

Creep recovery measurement of the lead C_i_-PBA and C_i_-PVA-BA gels was performed using 20 mm Peltier plate geometry on the ARG-2 rheometer at 25 °C with 5 Pa stress over 180 s. The recovery measurement was recorded for 320 s. Creep and recovery compliance and percent strain as a function of time were reported.

### 2.5. Physical Observations 

The addition of materials may cause evident changes in the flow and physical properties of the gel formulations. Hence, analyzing these physical changes along with the rheology are important parameters in gel characterization. For this, the physical properties such as the homogeneity, syringeability, and self-healing/spinnbarkeit behavior were analyzed. The syringeability was checked using 1 mL syringe with 15-gauge needle connected to Intramedic polyethylene tubing (Becton Dickinson and Company, Franklin Lakes, NJ, USA).

### 2.6. Osmolality Measurements 

The osmolality was analyzed using Vapro^®^ Model 5600, (Wescor, Inc., Logan, UT, USA), calibrated with osmolality standards of 100 and 290 mmol/kg. The gels were diluted with DI water to obtain the appropriate consistency in order to completely cover the sample disc. Once analyzed, the measured values were adjusted to the dilution factor and represented as an average of three runs (*n* = 3).

### 2.7. Fourier Transform Infrared Spectroscopy (FTIR)

In order to map the functional group interactions and cross-linking efficiency between the polymers in gels, Attenuated Total Reflectance-FTIR (ATR-FTIR) analysis was performed (Nicolet™ iS™ 10, Thermo Fisher Scientific, Waltham, MA, USA). For this, specific quantities of the materials were placed on sample holder and scanned (*n* = 10) in the range of 4000–525 cm^−1^.

### 2.8. Stability Analysis 

The selected gel formulations were tested for short-term stability under different temperature conditions to evaluate their appropriate storage conditions. Briefly, the formulations were stored in glass vials at 25 °C, 4 °C, and 37 °C for up to 9 days to observe any physicochemical (e.g., pH, homogeneity, color), rheological, or viscoelastic changes using the methods discussed above. 

### 2.9. In Vitro Sperm Functionality Testing 

**Figure 1:**
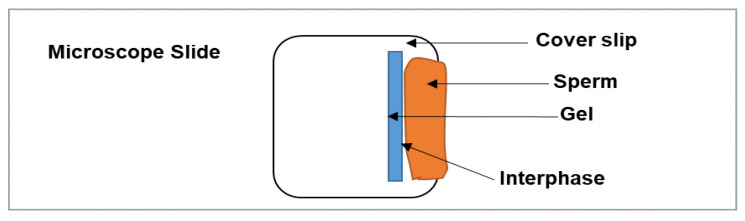
Schematic representation of the in vitro sperm penetration testing of the gels.

A physiologically relevant in vitro test method was developed using human or rabbit sperm to simulate testing of gel effectiveness as a physical sperm barrier. For the human sperm, volunteers donated semen under the EVMS Institutional Review Board (IRB) approved protocol #13-02-FB-0031. Rabbit sperm were collected under the Eastern Virginia Medical School (EVMS) Institutional Animal Care and Use Committee (IACUC) approved protocol # 19-022 (animal source and handling is provided under Section 2.10 below). Initially, the semen samples were analyzed on computer-assisted sperm analyzer (CASA) to evaluate the quality of the sperm, and sperm with ≥50% motility were used. Before testing, the samples were kept in a CO_2_ incubator at 37 °C for 30 min. The universal hydroxyethyl cellulose (HEC) placebo gel was used as a reference (negative control) [28], and the N-9 gel at 4% w/w was used as a positive control. In brief, the human or rabbit sperm sample was placed adjacent to the test samples (~100 µL) on a microscope slide (Figure 1). A coverslip with a mixture of Vaseline and glass beads applied on the 4 corners was slowly placed on top of the gel. About 50 µL of sperm sample was applied from one end of the cover slip until the sperm encountered the gel edges. The penetration of sperm passing through the sperm–gel interface was tracked at room temperature and then after 20 min incubation at 37 °C in a CO_2_ incubator (to ensure temperature-induced thinning of the gel did not compromise the gel’s barrier properties at body temperature). The distance (in µm) traveled by sperm that penetrated the gel was calculated from the point of contact of sperm with gel, referred to as interphase. The sperm penetration values are represented as mean ± SD of four different penetration sites on the same slide. The sperm penetration as well as spreading of gel under coverslip was completed using crossline reticules (Klarmann Rulings, Inc., Litchfield, NH, USA), fitted to the objective of a Nikon Eclipse E800 microscope. The images were captured using a CCD spot camera (Diagnostic Instruments, Inc., Sterling Heights, MI, USA). 

### 2.10. Rabbit Contraceptive Efficacy Test (RCET)

Based on the in vitro investigations, the lead gels were selected for contraceptive efficacy testing in rabbits. Sperm was collected from a colony of male New Zealand White Rabbits (Envigo Global services Inc., Denver, PA, USA), maintained in an animal housing facility at EVMS. Sperm was collected from male rabbits using an artificial vagina and analyzed in a computer assisted sperm analyzer (CASA). The semen samples that showed ≥50% motility were pooled and adjusted to a concentration of 50 million motile sperm/mL in medium containing Tyrode’s albumin lactate pyruvate (TALP) combined with 0.1% bovine serum albumin (BSA). Each animal was administered ~2 mL of the respective samples to the upper vagina via a syringe connected with a flexible catheter. After 15 min, the animals were artificially inseminated with ~0.5 mL of pooled rabbit sperm. Following insemination, human Chorionic Gonadotropin (hCG from Sigma, St. Louis, MO, USA) was applied at 0.25 mL/rabbit through the ear vein. Animals were then maintained for at least 11 days before euthanasia. At necropsy, the implantation sites were counted and compared, and the animals were defined as pregnant if any implantation sites were observed.

## 3. Results and Discussion

### 3.1. Rheological Characterization of PBA-SHA (Control) Gel

**Figure 2:**
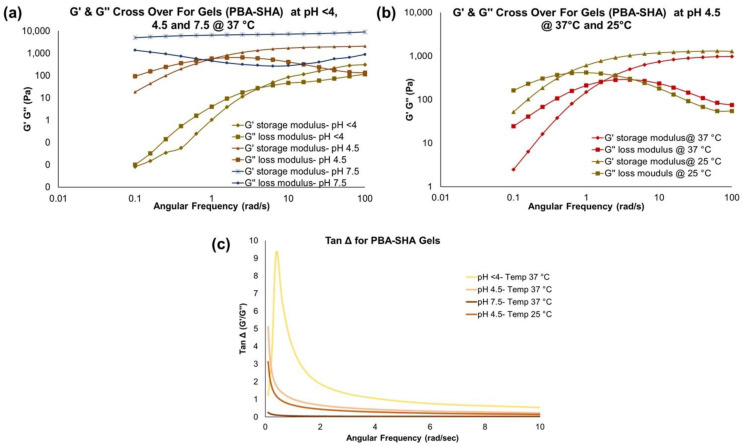
PBA-SHA gel rheology data: (**a**) pH-dependent (at <4, 4.5, and 7.5) and (**b**) temperature-dependent (at 25 and 37 °C) changes in the G′ and G″ modulus profiles at varying angular frequency. (**c**) Tan Δ plots confirming both temperature and pH affected the viscoelastic strength of the gel.

PBA-SHA gel was evaluated by dynamic rheology to verify that the rheological behavior is consistent with previously published results [9] and support its use as a reference control barrier gel in the studies described herein. As expected, the chemically cross-linked PBA-SHA gel showed frequency-dependent viscoelastic behavior that was both pH and temperature dependent. The crossover frequency (ω_c_, defined as the frequency at which G′ = G″) shifted from ≥1 rad/s at pH < 4 to 0.9 ± 0.2 rad/s (*n* =3) at pH 4.5 to loss of cross over for storage and loss modulus within the measured 0.1–100 rad/s range at basic pH (7–8) (Figure 2a), indicative of a shift from more liquid-like to more viscoelastic-like behavior and notably similar to previously published results in which ω_c_ < 0.01 rad/s (0.008 rad/s at pH 7.6) was observed [9]. The plateau storage modulus (G′_plateau_) also increased with pH, indicating a stronger cross-linked gel network. This increase in the crossover modulus was observed because of the strengthening of coordinate covalent bond formation between PBA and SHA with increase in pH. The gel formation and strengthening are reversible in nature and generates dynamically cross-linked gel networks [9]. PBA-SHA gel showed a temperature-dependent decrease in the crossover frequency and, to a lesser extent, G′_plateau_ (Figure 2b), indicating a gel softening or loss of elasticity, which is further consistent with previously published findings for the PBA-SHA gel and chemical crosslinking chemistry [9]. The Tan Δ plots (Figure 2c) of PBA-SHA gel also confirmed that both temperature and pH affected the viscoelastic strength of the gel.

### 3.2. Development of Physically Cross-Linked C_i_-PBA Gels

**Figure 3:**
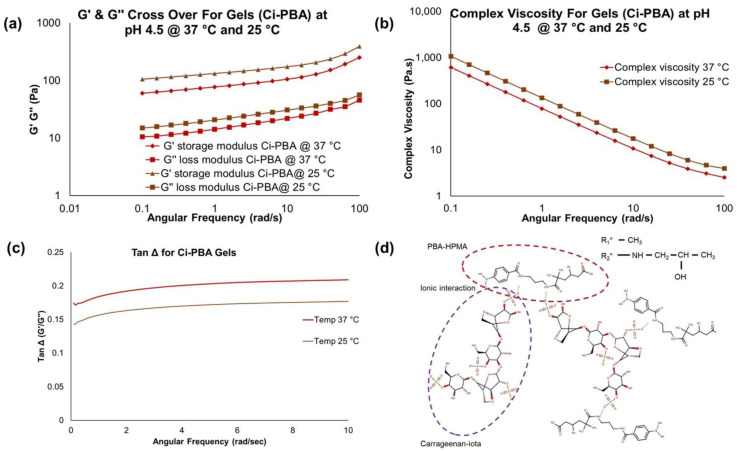
Physically cross-linked C_i_-PBA gel (Gel 2) rheology data at pH 4.5: (**a**) temperature-dependent (at 25 and 37 °C) changes in the G′ and G″ modulus profiles at varying angular frequency, (**b**) temperature-dependent (at 25 and 37 °C) changes in the complex viscosity, (**c**) Tan Δ plots, which confirming the gel was softer at 37 °C compared to 25 °C, and (**d**) ionic interaction between PBA and C_i_ polymers in forming the C_i_-PBA gel.

The physically cross-linked C_i_-PBA gels were prepared in acetate buffer using polymers at different concentrations (Table 1) to analyze the effect on viscoelastic behavior. In C_i_-PBA gels, on vaginal application at acidic pH, weak gel network was predominantly formed by the inherent gelling property of C_i_, but also via van der Waals, H-bonding, and hydrophobic interactions with PBA polymer. The presence of the sulfated groups in C_i_ polymer are esters of a very strong acid, which accumulate electronegative charge at acidic (3.5–4.5) or neutral to basic pH (7–8) and generates an ionic interaction with cationic amine functionality of the HPMA copolymer backbone of PBA polymer. These ionic interactions in addition to the above van der Waals, H-bonding, and hydrophobic interactions helped in forming a physically cross-linked C_i_-PBA gel at basic pH (7–8) (Figure 3d). The screening results showed that the polymeric ratios of PBA-HPMA and C_i_ were critical in developing a physically cross-linked gel, and increasing the PBA concentration formed a harder gel (Gels 1, 5, and 6) whereas, a higher relative concentration of C_i_ (Gel 1 (20 mg/mL)) created a harder and crumbly gel. As the relative concentration of C_i_ increased, the zero-shear-rate viscosity also increased, which showed that the addition of specific amounts of C_i_ affected the viscoelastic behavior of gels formed by ionic or chemical crosslinking interaction with PBA-HPMA polymers. Some air bubbles were also observed in the gels having a higher concentration of C_i_, specifically when made in larger volumes (≥25 mL), thus Gel 7 containing lower concentration of C_i_ (8 mg/mL) was also investigated.

#### 3.2.1. Frequency Sweep Rheology and Tan Δ Measurements for C_i_-PBA Gels

The G′ and G″ measurements from frequency sweep runs are important parameters to evaluate the viscoelastic behavior of the materials. For the gel formulations, the G′ is generally higher than G″ modulus, however, the values are dependent on the polymer concentration and pH, both influencing the cross-linking behavior [29,30]. The cervicovaginal fluid is shown to have a viscoelastic behavior [31,32] and its rheological properties could help in designing vaginal contraceptive gels. Frequency sweep studies with cervical mucus showed G′ > G″ across all frequencies tested, with G′ and G″ moduli values in the range of 23.4–13.6 Pa and 5.4–4.6 Pa, respectively [32], confirming its viscoelastic nature [32]. In our study, physically cross-linked C_i_-PBA gel (Gel 2) showed similar viscoelastic behavior (Figure 3a–c) to that of cervical mucus. Notably, the rheological profile for the C_i_-PBA gel did not reveal a crossover frequency as observed with the chemically cross-linked PBA-SHA gel, indicating that C_i_-PBA formulation was a physically cross-linked gel [21]. From the rheology data (Table 1), it seems that C_i_ plays a critical role and the gel was formed by an ionic interaction between C_i_ and PBA polymers. The presence of the sulfated functional groups in C_i_ polymer accumulate electronegative charge at acidic pH, which perhaps prevents the formation of diol-interaction of C_i_ with PBA [9], but alternatively generates an ionic interaction in forming a physically cross-linked gel (Figure 3d). 

**Figure 4:**
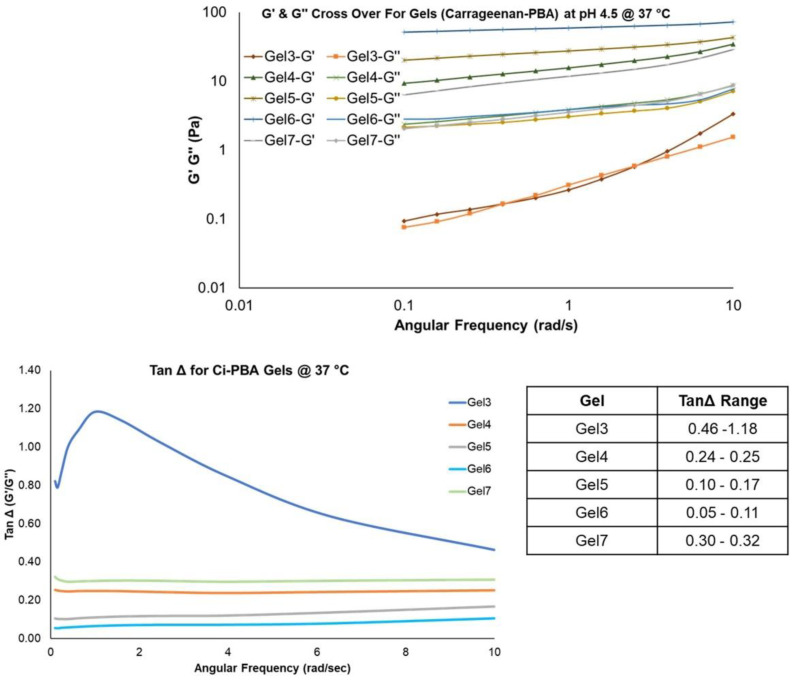
G′ and G″ overlays and Tan Δ values for C_i_-PBA gels. The inserted table shows the range of tan Δ values for the gels.

**Figure 5:**
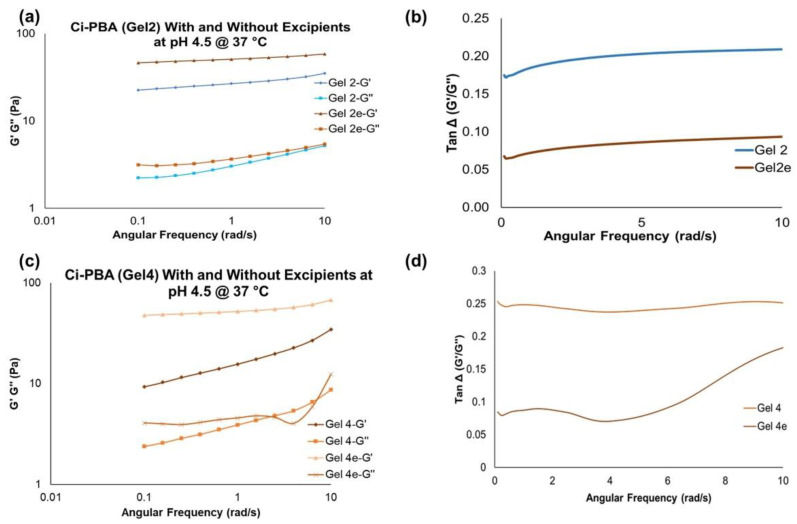
G′ and G″ overlay values and Tan Δ values for C_i_-PBA Gel 2 (**a**,**b**) and Gel 4 (**c**,**d**), respectively, (2e and 4e are gels with excipients). The values show that gels with excipients were relatively harder compared to the non-excipient formulations.

In comparing the behavior of C_i_-PBA gels of varying C_i_ and PBA-HPMA polymer concentrations, it was also observed (Table 1 data) that Gel 4 that had slightly less PBA concentration (30 mg/mL compared to 33.33 mg/mL in Gel 2) showed similar characteristics to Gel 2 in terms of rheology, syringeability and self-healing properties, and selected for further investigations. This also provided the information that a slight change in the PBA polymer concentration did not provide much influence on the gel rheology. However, Gel 3 with lower (5 mg/mL) and Gel 5 with relatively higher concentrations of C_i_ (20 mg/mL), Gel 6 with higher amount of PBA (50 mg/mL) and Gel 7 with relatively lower concentrations of both PBA (20 mg/mL) and C_i_ (8 mg/mL) (Table 1) were also tested for rheology to further investigate the effects of polymer concentrations. Frequency sweep measurements displayed a stepwise increase in the G′ and G″ values (Figure 4) with increase in C_i_ concentrations in the gels. Gel 6 showed the highest relative G′ and G″ values and was a harder gel, possibly due to a very high concentration of PBA [33]. The complex viscosity data from the frequency sweep measurements were also analyzed for obtaining the zero-shear-rate viscosity using Williamson model, which is commonly used in determining the viscosity of gels and fluids at low shear rates [34]. The correlation coefficient (r^2^) for the Williamson model was found to be >0.99. The increase in G′, G″, and zero-shear viscosity values suggested that the gel formulations could provide contraceptive effects by serving as a physical biomimetic plug to block sperm penetration. The tan Δ value for C_i_-PBA gels was in the range of 0.05–1.18, (Figure 4 and Figure 5) and decreased with increasing the C_i_ concentration. Gel 3 showed a tan Δ value of 1.18 (>1), which aligned with visible observation of its viscous nature, but Gel 2 (0.17–0.21), Gel 4 (0.24–0.25), and Gel 7 (0.30–0.32) showed tan Δ values and consistency close to that of the cervical mucus (0.27–0.38) [32] to provide a desirable syringeability and physical barrier properties in vitro and in vivo, and selected for further evaluation.

#### 3.2.2. Effect of Excipients on Viscoelastic Behavior of C_i_-PBA Gels

To make the final gel formulations for in vitro sperm functionality testing, methyl and propyl parabens at a combined concentration of 0.27% w/w (as preservatives) [22], and glycerol at 2.5% w/w (to dissolve the parabens and to enhance the syringeability and in vivo applicability of the gel) were added to Gel 2 and Gel 4 (called as Gel 2e and 4e, respectively, ‘e’ stands for the excipients). Rheological investigations showed that the addition of these excipients increased the viscoelastic behavior of the gel, also confirmed by an increase in the G′ and G″ values (Figure 5a,c). Moreover, the corresponding tan Δ value of ~0.1 in the presence of excipients also defined a thick gel formation (Figure 5b,d). 

### 3.3. Development of pH-Dependent Chemically Cross-Linked C_i_-PVA-BA Gels

**Figure 6:**
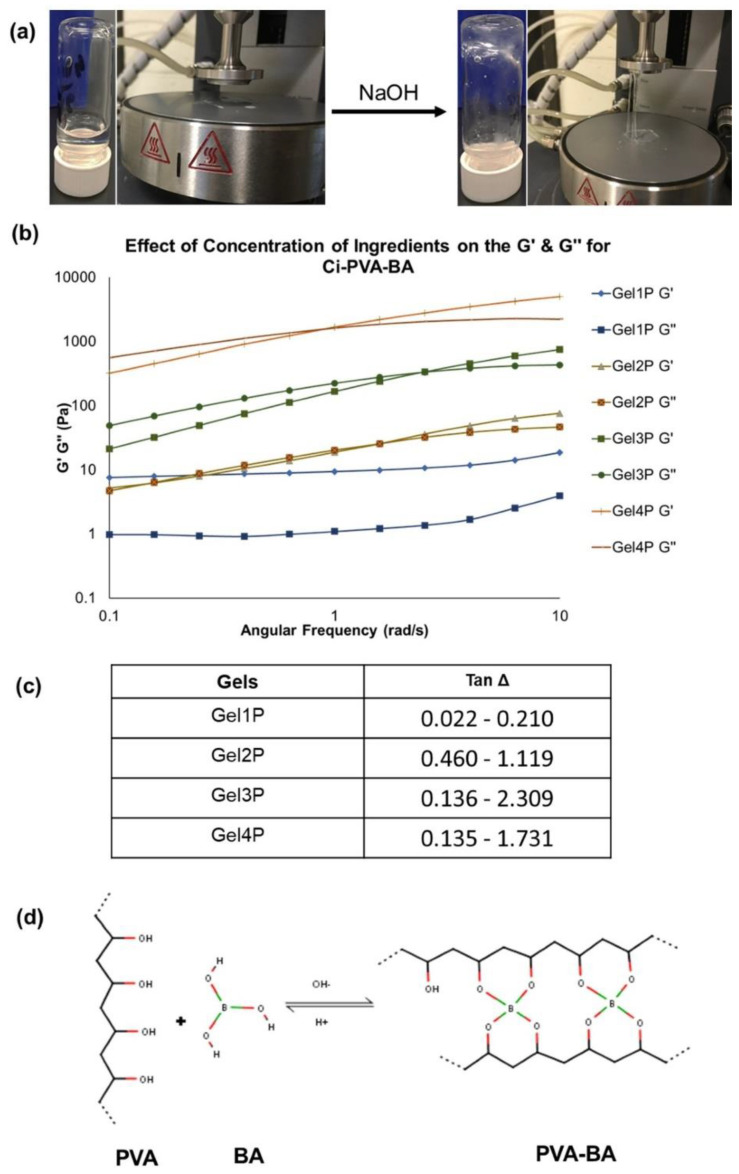
(**a**) Physical observations of Gel 4P (representative C_i_-PVA-BA gel) showing that it as a soft, weak, flowing gel at acidic pH of ~4–4.5 with no spinnbarkeit behavior whereas, increasing the pH to 7–8 generated a chemically cross-linked system with characteristic spinnbarkeit. (**b**) Effect of polymers and cross-linker concentrations on the conversion of physical to chemically cross-linked gels after adjusting the pH from acidic to 7–8, analyzed through G′ and G″ modulus crossover frequency measurements. (**c**) Tan Δ values for Gel 1P to Gel 4P. (**d**) Representative cross-linking reaction between PVA and BA under acidic and basic pH conditions.

To develop a chemically cross-linked C_i_-PVA-BA gel, and using all off-the-shelf polymers and excipients, C_i_ polymer was added as a gelling (thickening) agent at acidic pH [35], whereas PVA and BA were used to generate a chemically cross-linked hydrogel at basic pH [36,37]. In the literature, PVA is also shown to form physically cross-linked hydrogels with polysaccharides such as chitosan and carrageenan for wound healing and drug delivery applications [14,38,39]. In forming a pH-sensitive chemically cross-linked C_i_-PVA-BA gel, initially the weakly cross-linked transient network was formed on vaginal application at acidic pH, predominated by the inherent gelling property of C_i_ and somewhat to its interaction with the PVA polymer. At this stage, there was probably no cross-linking or interaction of BA (un-ionized at acidic pH) with the hydroxyl groups of PVA. However, when the pH was increased to basic, borate ions were generated from BA and assisted in its rapid cross-linking (due to borate-diol cross-linking) with PVA via forming a di-diol functionality [40] or hydrogen bonds [41], both helped in creating a pH-sensitive chemically densely cross-linked gel (Figure 6d).

The C_i_-PVA-BA gels were prepared by varying the concentrations of the components (Table 2) to investigate the conversion of a physically cross-linked system at acidic pH to chemically cross-linked gel at neutral to basic pH to act as a physical barrier to vaginal sperm transport. The rationale was to design a formulation that can modulate its properties in forming a viscoelastic, weakly cross-linked, transient network on vaginal application (acidic pH) to a more elastic, densely cross-linked network at increased pH that may serve as a physical barrier to motile sperm. It was observed that increasing the concentrations of PVA and BA increased the G′ and G″ values (Table 2), whereas gels with low C_i_ amounts (5 mg/mL) were relatively fluidic. The cross-linking data for the gels under acidic and basic conditions were also fitted (r^2^ > 0.99) to the Williamson model to determine the zero-shear-rate viscosity of gels at low shear rates (Table 3).

As shown in Figure 6a,b, subsequent increases in the G′ and G″ values of C_i_-PVA-BA gels at basic pH indicated an increased viscoelasticity and performance of a chemically cross-linked gel with characteristic spinnbarkeit behavior. Gels 1P and 5P, having the least amounts of PVA and BA showed that the cross-linking disappeared and exhibited only physically formed gels, primarily attributed to gelling properties of the C_i_ polymer. However, the gels with KCl (2P, 3P and 4P) and without KCl (6P and 7P) exhibited pH-dependent transition from a physical gel to chemically cross-linked gels. Since BA alone did not cross-link with PVA (mixture of BA and PVA was liquid at acidic pH), to compensate for this behavior, C_i_ was added to enhance the viscosity and potentially the in vivo retention of the gel. However, the cross-linking of borate ions (formed after BA was exposed to a basic pH conditions) and PVA occurred rapidly via forming a di-diol bond (Figure 6d), which indicated a chemically cross-linking behavior of the gel at basic pH. 

Tan **Δ** explains the capability of a material or gel to dampen the applied strain that is at equilibrium between G′ and G″. If tan Δ is high,gels are extremely viscous compared to gel with low tan Δ (suggesting that gels are more elastic in nature) [37]. It was observed that C_i_-PVA-BA gels have relatively high tan Δ (>1) (Figure 6c) at basic pH compared to C_i_-PBA gels, which indicated that PVA-BA crosslinking caused gels to be more viscous in nature, also confirmed by its distinct spinnbarkeit behavior (Figure 6a). It was also observed that Gel 1P with lower PVA/BA concentration (10 mg/mL and 5 mg/mL, respectively) had high elastic behavior owing to the C_i_ component of the gel. With increase in PVA/BA concentration increased the cross modulus and the viscosity of the gels (Gel 2P-4P) (Figure 6b,c). The data provided in Table 2 and Figure 6 reflected that KCl aided in the internal cross-linking of functional groups of C_i_ polymer [21], but created a cloudy gel at acidic pH (possibly due to KCl precipitation) [42], which was not seen in the absence of KCl (Gels 5P, 6P, and 7P). Hence, to avoid the precipitation generated from KCl, but to compensate for the KCl-induced cross-linking and thickening, higher concentrations of C_i_ were tested (Gels 6P (9 mg/mL) and 7P (9.5 mg/mL)). Gels 6P and 7P both showed better syringeability and were not liquid at basic pH, but Gel 7P showed clear transitioning from physical to chemical-crosslinking behavior when the pH was changed from acidic to basic and tested further. 

**Figure 7:**
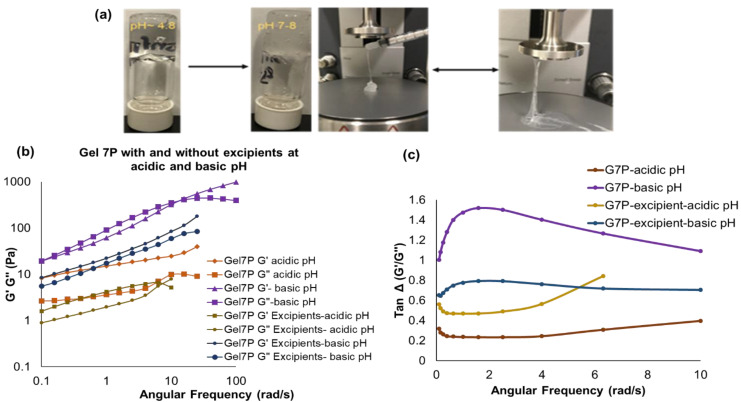
(**a**) Physical observations with C_i_-PVA-BA gel 7P exhibiting a soft syringeable (no spinnbarkeit) characteristic at acidic pH (~4.8), but a thicker viscoelastic gel with spinnbarkeit behavior was observed when the pH was increased to 7–8. (**b**) Frequency sweep results of Gel 7P with and without excipients at acidic and basic pH. (**c**) Tan Δ plots for gel 7P under acidic and basic conditions.

Physical observations with Gel 7P exhibited a soft syringeable (no spinnbarkeit) gel characteristic at acidic pH (~4.8), but a thicker (higher viscosity) gel with spinnbarkeit behavior was observed when the pH was changed to 7–8 (Figure 7a). The frequency sweep data (Figure 7b) showed that the zero-shear viscosity was increased at higher pH, which confirmed that the C_i_-PVA-BA gel became viscoelastic due to pH-dependent transition to a chemically cross-linked gel. As discussed previously, tan Δ plot (Figure 7c) revealed that Gel 7P at basic pH was found to viscous compared to Gel 7P at acidic pH. However, addition of excipient (containing glycerol) prevented crosslinking creation between PVA-BA to provide optimum viscoelasticity (Tan Δ~0.087–0.793) for further biological evaluations [43]. Based on the screening of gel formulations, physicochemical characterization, appropriate syringeability, and dynamic rheology results, C_i_-PVA-BA Gel 7P (30 mg/mL of PVA, 10 mg/mL of BA, and 9.5 mg/mL of C_i_, pH of ~4.8) was selected for in vitro functionality and rheology testing in the presence of excipients. 

#### Rheology of C_i_-PVA-BA Gel (7P) in the Presence of Excipients

Similar to the C_i_-PBA gel, methyl and propyl parabens in glycerol were added to the C_i_-PVA-BA gel. The gel without excipients showed physical-to-chemical cross-linking transition at the pH of 7–8, but the gel with excipients did not show any crossover frequency (cross-linking), nevertheless with prominent physical changes (Figure 7b). The disappearance of cross-linking of PVA with BA, we hypothesize, may have been because of an interaction between glycerol and BA to form sodium bis (glycerol) borates bi-product [43,44,45]. This may have resulted in insufficient quantities of BA in the sample to produce a strong chemically cross-linked gel with PVA, but with prominent physical changes since there was an increase in the G′ and G″ values for the gels with excipient at basic pH compared to acidic pH. These data showed that although glycerol is commonly used as humectant/lubricant and solvent for preservatives, its concentration plays a critical role and needs to be optimized on a case-by-case basis.

### 3.4. Creep Recovery Analysis of Ci-PBA and Ci-PVA-BA Gels

The creep recovery (quantitative) and visual (qualitative) testing of the lead gels (Ci-PBA Gel 2 and Gel 7 and Ci-PVA-BA Gel 7P) was performed to confirm their self-healing and mechanical properties (Supplementary File Data and Figures S1 and S2). Data confirmed that gels were able to recover back to their state of origin after removal of the stress after attaining deformation, indicating their self-healing and viscoelastic characteristics.

### 3.5. Osmolality Measurements of C_i_-PBA and C_i_-PVA-BA Gels

The osmolality of vaginal formulations should not be too high to minimize the risk of vaginal epithelial damage [46]. Specific recommendations have been proposed with desirable values of ≤380 mOsm/kg; however, most of the over-the-counter vaginal lubricants exceed this value and WHO recommended acceptable values as high as 1200 mOsm/kg on an interim basis [47,48]. In this study, the osmolality values of C_i_-PBA and C_i_-PVA-BA gels were in the range of ~350–500 mOsm/kg, which was well below the 1200 mOsm/kg, acceptable for intravaginal application.

### 3.6. FTIR Analysis of C_i_-PBA and C_i_-PVA-BA Gels

**Figure 8:**
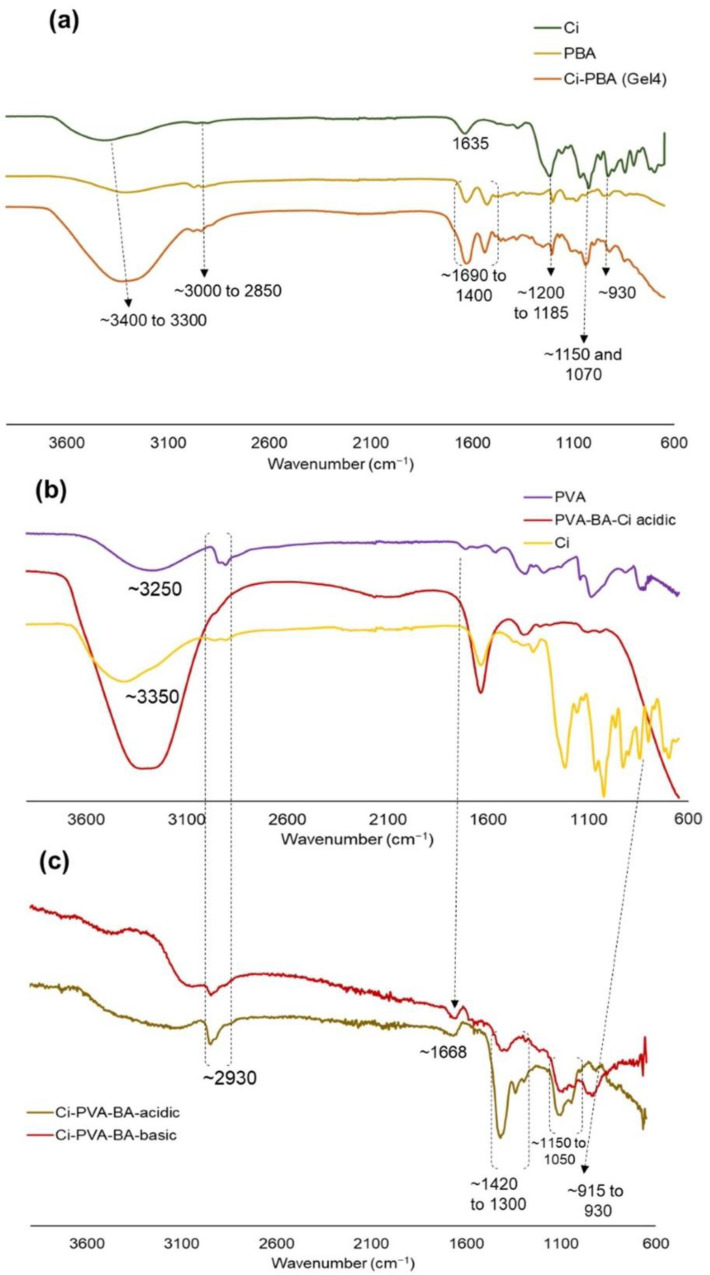
FTIR spectra of: (**a**) C_i_-PBA Gel 4 and the native PBA and C_i_ polymers, and (**b**) C_i_-PVA-BA (Gel 7P) and native PVA and C_i_ polymers. (**c**) FTIR spectra of Gel 7P with subtracting the aqueous buffer peaks before and after the pH adjustments from acidic to basic conditions. The *x*-axis and *y*-axis represent percentage (%) transmittance and wavenumber (cm^−1^), respectively.

To analyze the functional group interactions between polymers, FTIR analysis was performed for C_i_-PBA and C_i_-PVA-BA gels and native polymers (C_i_, PBA, and PVA) as controls (Figure 8). The C_i_-PBA gel showed signature peaks of −CH− and –OH stretching in the range of ~3000 to 2850 cm^−1^ and ~3400 to 3300 cm^−1^, respectively, which were present in the control samples. There were also doublets (1690 to 1400 cm^−1^) from the −C=O group of the amide functional group of the PBA polymer (Figure 8a). The gels also showed water deformation peak at ~1635 cm^−1^; ester sulfate O=S=O symmetric vibrations at ~1200 to 1185 cm^−1^; C−O stretch at ~1150 and 1070 cm^−1^; C−O−C peaks at ~930 cm^−1^; and −O−SO_3_ stretching vibration at ~850 and 800 cm^−1^ of the C_i_ polymer [49]. The presence of multiple signature signals in the gels and of the control materials, suggested that the interaction between the polymers was a non-covalent type, which was confirmed by the rheology data that these gels were physically cross-linked through ionic interactions. In the chemically cross-linked C_i_-PVA-BA gel (Gel 7P), the spectra revealed typical bands of C_i_ (discussed above) and PVA (at ~839, 917, and 1089 cm^−1^, attributed to C−C bending, −CH_2_ rocking, and ester C−O−C bending from the acetyl groups) polymers (Figure 8b) [50]. For both PVA and C_i_, strong stretching vibrations were observed at ~3250 and 3350 cm^−1^, attributed to the −OH groups, and signature −CH− stretching in the range of 2930 cm^−1^. Noticeably, PVA polymer showed a doublet (~1420 to 1300 cm^−1^) for the OH−C−OH group at acidic pH, which was almost disappeared at neutral to basic pH due to cross-linking between PVA and BA (Figure 8c) [51,52]. The cross-linking of PVA with BA was also confirmed with reduction in the intensity of (C−O)−C−OH)) group peak at 1150 to 1050 cm^−1^ [51,52]. The gel under acidic pH showed sulfate functionality of the C_i_ polymer at ~850 and 800 cm^−1^, but at basic pH, this was slightly shifted (~915 to 930 cm^−1^), possibly due to resonance generated from the cross-linking reaction.

### 3.7. Stability Testing

**Figure 9:**
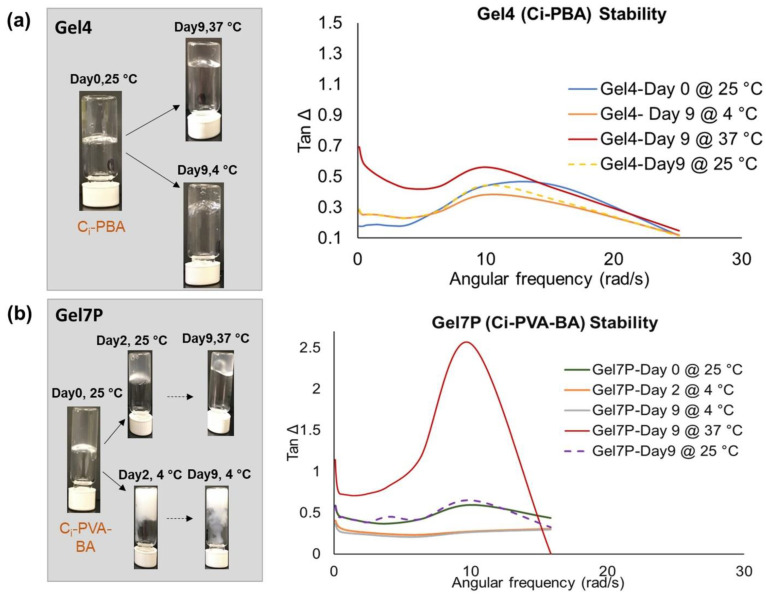
Tan Δ values and physical changes observed for: (**a**) C_i_-PBA gel (Gel 4) and (**b**) C_i_-PVA-BA gel (Gel 7P) stored at 25 °C, 4 °C, and 37 °C for up to 9 days.

The gel formulations were tested for their rheology and physical changes under different temperatures. Physical observations (Figure 9a) suggested that C_i_-PBA gel was stable at 25 °C and 4 °C (tested up to 9 days) with no changes in the homogeneity, pH, or color. However, being a physical gel, it became softer and fluidic at 37 °C, which is a common phenomenon for gels at higher temperature, and suggested that the C_i_-PBA gel should be best stored at ≤25 °C. In the case of C_i_-PVA-BA gel, at 4 °C the gel turned milky white after day 2 (Figure 9b). Although it remained clear at 25 °C and 37 °C up to day 9, but like the C_i_-PBA gels, it became softer and fluidic at 37 °C. Hence, the best storage condition for the C_i_-PVA-BA gel would be the ambient temperature (~25 °C). These physical observations were also supported by the tan Δ plots (Figure 9a,b) of the rheological frequency sweep runs, which confirmed that the gels were relatively softer and fluidic at 37 °C (tan Δ > 1) compared to at 4 °C or 25 °C with tan Δ was < 1.

### 3.8. In Vitro Sperm Functionality Testing (Sperm Motility, Viability, and Penetration)

Based on the screening of prototype gel formulations, the lead gels with promising rheological data demonstrating gelling capability at low concentrations and at pH 4–5 selected for in vitro sperm functionality testing are summarized in Table 4. The C_i_-PBA Gels 2 and 4 were tested because both showed similar rheology, syringeability, and self-healing properties close to cervical mucus. In the case of C_i_-PVA-BA Gel, based on the rheology data, Gel 7P was selected for in vitro functionality testing. Results showed that the C_i_-PBA Gel 2 provided a strong sperm penetration inhibition (compared to the controls) and created a barrier at the sperm-gel interface at room temperature with suitable syringeability and spinnbarkeit behavior. There was no pH-dependent thickening observed for all the C_i_-PBA gels at basic pH (~7–8) of sperm samples, which confirmed their design of a physically cross-linked gel. The C_i_-PVA-BA Gel 7P also created a barrier at the sperm-gel interface with suitable syringeability and spinnbarkeit behavior, but the gel provided pH-sensitive thickening (increased cross-linking) when exposed to a higher (basic) pH of sperm samples. While the universal HEC placebo gel as a negative control allowed the sperm to rapidly penetrate through the gel, the N-9 4% gel (positive control) did not provide any penetration, but all the sperm died or disintegrated upon contact with N-9. 

### 3.9. In Vivo Contraceptive Efficacy Testing of the Gels

The RCET study included four animal groups: sham control, gel samples, HEC gel (negative control), and N-9 (positive control). The in vitro sperm penetration results confirmed that C_i_-PBA Gel 2 created a strong barrier at the sperm-gel interface with suitable syringeability and spinnbarkeit behavior, supporting its confirmatory RCET in rabbits. The C_i_-PBA Gel 7, which showed no/minimal bubble formation was also tested in RCET. Two separate RCET studies were conducted and a summary of both of these studies are provided in Table 5. The results demonstrated a contraceptive efficacy of 60% for C_i_-PBA Gel 2 and 40% for C_i_-PBA Gel 7. Though Gel 2 showed a strong in vitro sperm penetration barrier, it did not show a 100% contraceptive efficacy in rabbits, possibly because the in vitro barrier was not sufficiently effective in in vivo efficacy testing using the rabbit model. Another possible reason for not achieving a complete contraceptive efficacy could be an inadequate distribution of the gel samples to cover the cervical os (noting rabbits have two cervices, each with a separate uterine horn). 

## 4. Conclusions

This study explored the development of novel vaginal contraceptive gel formulations that provide a physical barrier to sperm transport. Two types of formulations, physically cross-linked C_i_-PBA and pH-shifting physically/chemically cross-linked C_i_-PVA-BA gels, were designed and characterized for their rheology, physicochemical properties, and in vitro sperm-penetration-blocking characteristics. The physically cross-linked C_i_-PBA gels were targeted to remain in the vagina after application and in the presence of seminal fluid to block sperm transport. The pH-sensitive C_i_-PVA-BA gels were designed to transition from a more fluid-like, physically cross-linked gel at low vaginal pH to a densely chemically cross-linked gel that may act as a physical (mesh) barrier to sperm when exposed to the higher pH of seminal fluid. In vitro results suggested that the polymer concentrations, pH, and excipients affected the rheology and spinnbarkeit behavior in both the gel formulations. Based on the screening data, lead C_i_-PBA and C_i_-PVA-BA gels were tested for in vitro sperm functionality, which confirmed that the gels provided sperm blocking properties to support their further in vivo testing. However, when C_i_-PBA gels were further evaluated in a rabbit contraceptive efficacy model, only partial protection was observed. Nonetheless, the in vitro and in vivo results together provide initial proof-of-concept that the C_i_-PBA gels are capable of providing sperm blocking properties and further formulation optimization is needed to enhance their barrier contraceptive efficacy.

## Figures and Tables

**Table 1:** Concentrations of C_i_ and PBA-functionalized polymers, rheological characterizations, and physical observations of C_i_-PBA gels. The G′_max_ and G″_max_ values were measured at 25 °C and marked as maximum values obtained for the scans in the range of 1 to 10 rad/s.

Gel	C_i_ (mg/mL)	PBA (mg/mL)	Williamson Model (r^2^)	Zero Shear Rate Viscosity (Pa. S)	Physical Properties	G′_max_ (Pa)	G″_max_ (Pa)
1	20	33.33	N.D. *	N.D.	Crumbly, hard, non-syringeable	173.56	30.73
2	10	33.33	N.D.	N.D.	Soft, syringeable with self-healing property	43.48	5.70
3	5	30	N.D.	N.D.	Fluidic, no gel behavior	3.33	1.54
4	10	30	0.98	125,107	Soft, syringeable	34.40	8.65
5	20	30	0.99	163,155	Hard but syringeable with self-healing property	42.84	7.17
6	10	50	0.99	354,482	Relatively hard with some syringeability	72.22	7.63
7	8	20	0.99	103,782	Extremely soft, syringeable	28.67	8.81

* N.D.: Not defined (gels did not fit into Williamson model to provide zero-shear rate viscosity).

**Table 2:** Final concentrations of the components, and rheological and physical characterizations results of C_i_-PVA-BA gels. The G′_max_ and G″_max_ measurements were made at 25 °C after increasing the pH to 7–8.

Gel	C_i_ (mg/mL)	PVA (mg/mL)	BA (mg/mL)	KCl (mg/mL)	Cross-linking	G′_max_ (Pa)	G″_max_ (Pa)	ω_c_ (rad/s)	Modulus at Crossover (Pa)
1P	5	10	5	2.5	No	18.73	3.93	NA	NA
2P	5	20	10	2.5	Weak	75.77	46.32	39.03	46.17
3P	5	30	15	2.5	Yes	752.13	431.05	2.44	330.59
4P	6	30	15	2.5	Yes	5020.03	2294.13	0.90	1570.58
5P	5	10	5	-	No	10.14	4.39	NA	NA
6P	9	30	10	-	Yes	175.97	125.05	9.49	122.59
7P	9.5	30	10	-	Yes	321.93	444.94	13.42	388.11

**Table 3:** C_i_-PVA-BA gels showed pH-dependent changes in the zero-shear rate viscosity.

Gel	Acidic pH		Basic pH	
	Zero-Shear Rate Viscosity (Pa·S)	Williamson r^2^ Value	Zero-Shear Rate Viscosity (Pa·S)	Williamson r^2^ Value
2P	2.99	0.93	44.39	0.99
3P	8.46	0.93	448.12	0.99
4P	50.67	0.99	4964.79	0.99
6P	2.23	0.94	41.59	0.99
7P	19.16	0.98	161.85	0.98

**Table 4:** In vitro sperm penetration testing results of C_i_-PBA and C_i_-PVA-BA gels and controls.

Samples *	Polymer Composition and Gel pH	Approximate Sperm Penetration Distance (µm) **	
		0 min	20 min
C_i_-PBA (Gel 2)	33.33 mg/mL of PBA, 10 mg/mL of C_i_, pH~4.4	0 ± 0	0 ± 0
C_i_-PBA (Gel 4)	30 mg/mL of PBA, 10 mg/mL of C_i_, pH~4.4	140 ± 3.5	390 ± 29.2
C_i_-PVA-BA (Gel 7P)	30 mg/mL of PVA, 9.5 mg/mL of C_i_, 10 mg/mL of BA, pH~4.8	150 ± 11.2	350 ± 35.4
HEC gel	2.7% w/w, pH~4.4	>500 µm	Spread all over
N-9 gel	4% w/w	0 ± 0 ***	Not applicable

* In vitro testing was conducted using human sperm. ** The sperm penetration distance was represented as mean ± SD of four different penetration sites on the same slide. *** The sperm were immotile, possibly dead due to the membrane-disrupting effect of N-9.

**Table 5:** Summary of RCET Study Results of Gels (2 mL of each samples used).

Treatment Arms	RCET # 1	RCET # 2	Summary of Two RCET Studies
	# Pregnant/Total (% Contraceptive Efficacy)	# Pregnant/Total (% Contraceptive Efficacy)	# Pregnant/Total (% Contraceptive Efficacy)
Sham control	3/3 (0%)	3/3 (0%)	6/6 (0%)
HEC gel (2.7% w/w)	4/5 (20%)	4/4 (0%)	8/9 (~11%)
N-9 gel (4% w/w)	0/2 (100%)	0/2 (100%)	0/4 (100%)
C_i_-PBA (Gel 2)	2/5 (60%)	Not applicable	2/5 (60%)
C_i_-PBA (Gel 7)	Not applicable	3/5 (40%)	3/5 (40%)

## Data Availability

The data presented in this study are available within the article or supplementary material.

## Supplementary Materials

The following supporting information can be downloaded at: https://www.mdpi.com/article/10.3390/polym14091728/s1, Figure S1: Qualitative observation for self-healing nature of Ci-PBA Gel 2 (**a**,**b**) and Gel7 (**c**,**d**). Fluidic nature of the gels helped it to self-heal exhibited by mechanical push with spatula. (**e**) Demonstrate self-healing nature of Ci-PVA-BA at acidic pH; Figure S2: Creep-recovery data for gels at 25°C. (**a**) Relative % strain v/s. time (seconds) plot for Ci-PBA Gel 2 and Gel 7 at acidic pH. (**b**,**c**) Creep and recovery compliance confirming viscoelastic and self-healing nature of Ci-PBA gels. (**d**) Creep-recovery profile (in terms of % strain) for Gel 7P under acidic and basic conditions showing self-healing nature upon removal of the stress. (**e**,**f**) Creep and recovery compliance confirming the viscoelastic nature of Ci-PVA-BA gel at both acidic and basic pH. References [53,54] are cited in the supplementary materials.
