# Supplementary material for: Development and Preclinical Investigation of Physically Cross-Linked and pH-Sensitive Polymeric Gels as Potential Vaginal Contraceptives
Source: Polymers (Basel). 2022 Apr 23;14(9):1728. doi: 10.3390/polym14091728 (PMC9101208; doi:10.3390/polym14091728)
Supplement: Supplementary file 1 [file polymers-14-01728-s001.zip › polymers-1641803-supplementary.pdf]

Supplementary Material

Development and Preclinical Investigation of Physically Cross-linked and pH-sensitive Polymeric Gels as Potential Vaginal Contraceptives

Ankit Rochani [1], Vivek Agrahari [2], Neelima Chandra [2], Onkar N. Singh [2], Timothy J. McCormick [2], Gustavo F. Doncel [2], Meredith R. Clark [2] and Gagan Kaushal [1,*]

[1] Department of Pharmaceutical Science, Thomas Jefferson University, Philadelphia, PA, USA
[2] CONRAD, Eastern Virginia Medical School, Norfolk, VA, USA

Creep Recovery Testing of the Gels

The viscoelasticity and self-healing of the gels was analyzed using visual observation (**Figure S1**) and creep recovery tests (**Figure S2**), including time-dependent creep and recovery compliance (observed strain/applied stress ratio) analyses [1–2]. For the physically cross-linked C_i-PBA Gel 2 (10 mg/mL C_i, 33.33 mg/mL PBA) and Gel 7 (8 mg/mL C_i, 20 mg/mL PBA), creep-recovery results demonstrated both gels were viscoelastic and self-healing **(Figures S1a-d and S2a-e**). Comparatively, Gel 2 required ~2-times lesser % strain to attain complete deformation and creep compliance was nearly 2-fold lower than Gel 7, indicating its comparatively stronger, more elastic nature. The recovery compliance of Gel 2 was almost the same as that of the creep compliance, indicating the gel fully recovered to its original state upon removing the stress, further confirming its self-healing behavior. For Gel 7, there was a significant difference between creep and recovery compliance, which was likely because of its relatively viscous behavior.

For pH-shifting cross-linked C_i-PVA-BA Gel 7P, creep-recovery results (**Figure S2d-f**) were similar to C_i-PBA Gel 2 at acidic pH. At basic pH, the creep-recovery results

demonstrate a viscoelastic profile with reduced instantaneous strain, increased creep strain over time, and yet similar recovery compliance as compared to the gel's acidic counterpart. Together, the results confirm the gel's self-healing nature (also observed visually, **Figure S1e**) and suggests an increased complexity of this formulation's pH-dependent viscoelastic behavior.

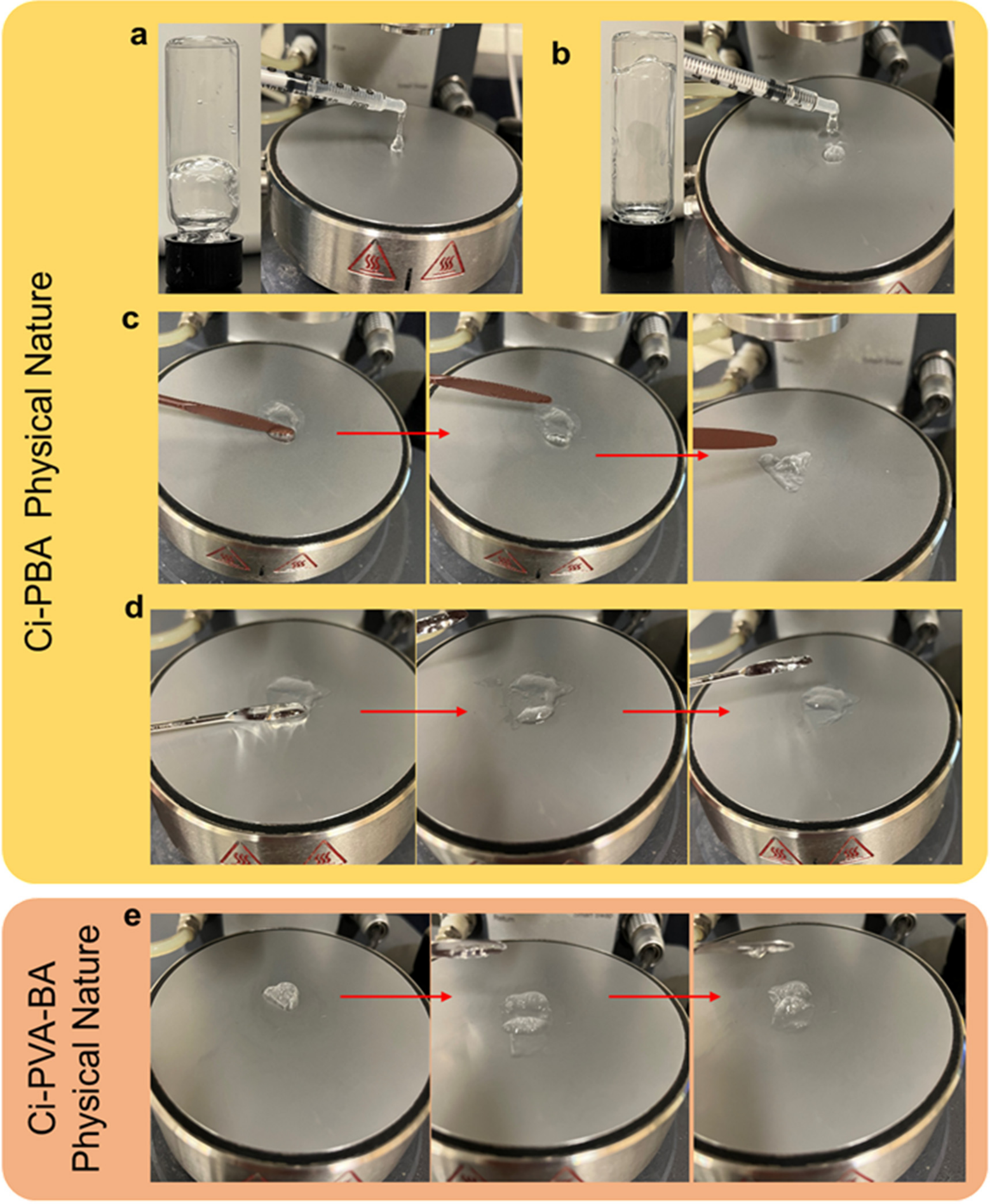

Figure S1: Qualitative observation for self-healing nature of C_i-PBA Gel 2 **(a and b)** and Gel7 **(c and d)**. Fluidic nature of the gels helped it to self-heal exhibited by mechanical push with spatula. **(e)** Demonstrate self-healing nature of Ci-PVA-BA at acidic pH.

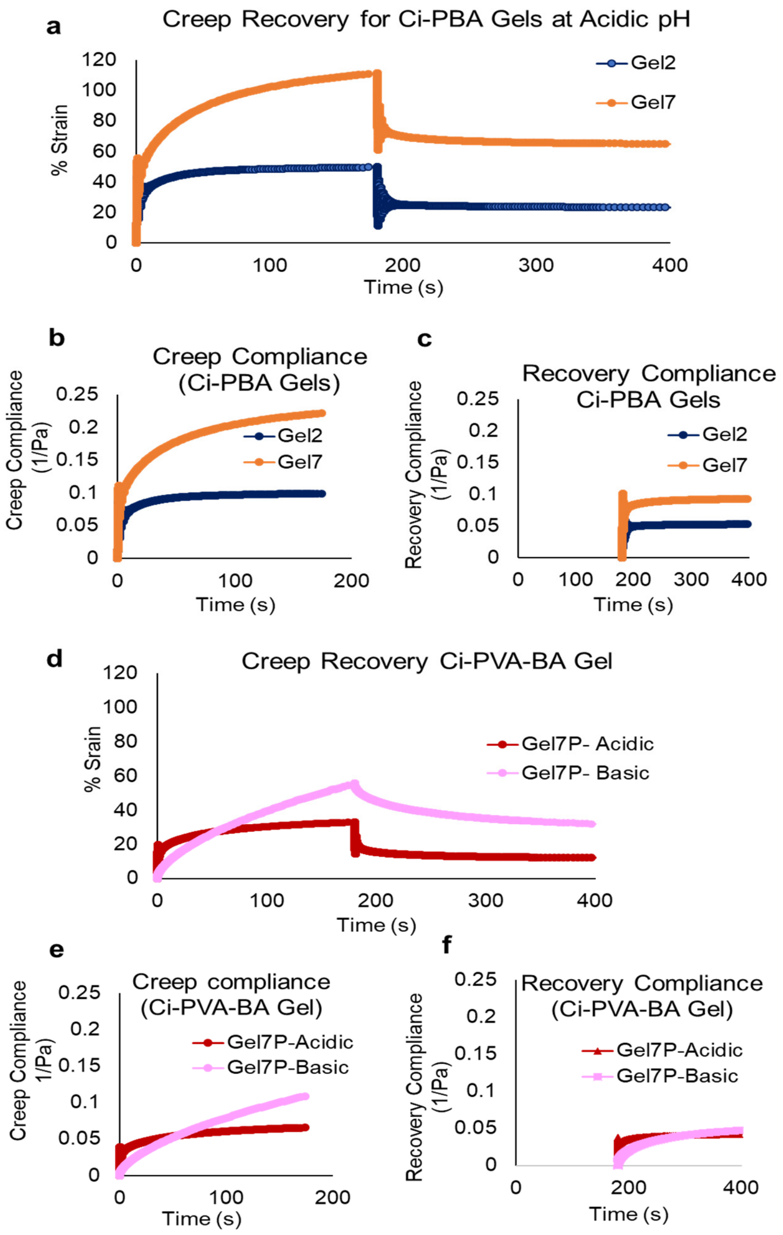

Figure S2: Creep-recovery data for gels at 25°C. **(a)** Relative % strain v/s. time (seconds) plot for C_i-PBA Gel 2 and Gel 7 at acidic pH. **(b and c)** Creep and recovery compliance confirming viscoelastic and self-healing nature of Ci-PBA gels. **(d)** Creep-recovery profile (in terms of % strain) for Gel 7P under acidic and basic conditions showing self-healing nature upon removal of the stress. **(e and f)** Creep and recovery compliance confirming the viscoelastic nature of C_i-PVA-BA gel at both acidic and basic pH.

Reference:

1. Kim, J.R. Laboratory development of a constitutive model for visco-elasto-plastic materials. *KSCE Journal of Civil Engineering* **1999**, *3*, 261-271, doi:10.1007/BF02823812.
2. Available online: https://www.tainstruments.com/wp-content/uploads/Boston-Rheology-Training-2019.pdf (accessed on 1 March 2022).
